# Olive Phenolics as c-Met Inhibitors: (-)-Oleocanthal Attenuates Cell Proliferation, Invasiveness, and Tumor Growth in Breast Cancer Models

**DOI:** 10.1371/journal.pone.0097622

**Published:** 2014-05-21

**Authors:** Mohamed R. Akl, Nehad M. Ayoub, Mohamed M. Mohyeldin, Belnaser A. Busnena, Ahmed I. Foudah, Yong-Yu Liu, Khalid A. EI Sayed

**Affiliations:** 1 Department of Basic Pharmaceutical Sciences, School of Pharmacy, University of Louisiana at Monroe, Monroe, Louisiana, United States of America; 2 Department of Clinical Pharmacy, Faculty of Pharmacy, Jordan University of Science and Technology, Irbid, Jordan; Florida International University, United States of America

## Abstract

Dysregulation of the Hepatocyte growth factor (HGF)/c-Met signaling axis upregulates diverse tumor cell functions, including cell proliferation, survival, scattering and motility, epithelial-to-mesenchymal transition (EMT), angiogenesis, invasion, and metastasis. (-)-Oleocanthal is a naturally occurring secoiridoid from extra-virgin olive oil, which showed antiproliferative and antimigratory activity against different cancer cell lines. The aim of this study was to characterize the intracellular mechanisms involved in mediating the anticancer effects of (-)-oleocanthal treatment and the potential involvement of c-Met receptor signaling components in breast cancer. Results showed that (-)-oleocanthal inhibits the growth of human breast cancer cell lines MDA-MB-231, MCF-7 and BT-474 while similar treatment doses were found to have no effect on normal human MCF10A cell growth. In addition, (-)-oleocanthal treatment caused a dose-dependent inhibition of HGF-induced cell migration, invasion and G1/S cell cycle progression in breast cancer cell lines. Moreover, (-)-oleocanthal treatment effects were found to be mediated via inhibition of HGF-induced c-Met activation and its downstream mitogenic signaling pathways. This growth inhibitory effect is associated with blockade of EMT and reduction in cellular motility. Further results from *in vivo* studies showed that (-)-oleocanthal treatment suppressed tumor cell growth in an orthotopic model of breast cancer in athymic nude mice. Collectively, the findings of this study suggest that (-)-oleocanthal is a promising dietary supplement lead with potential for therapeutic use to control malignancies with aberrant c-Met activity.

## Introduction

About 1 in 8 (12%) women in the US will develop invasive breast cancer during their lifetime [1]. The chance that breast cancer will be responsible for a woman’s death is about 1 in 36 (about 3%). The American Cancer Society estimated that about 232,340 new cases of invasive breast cancer will be diagnosed in women and about 39,620 women will die from breast cancer in the US in 2013 despite significant advances in detection and treatment [1]. Current chemotherapeutic treatments are usually not completely selective for carcinogenic cells and often induce significant cytotoxic effects on normal tissues, resulting in a decreased quality of life for cancer patients. Clearly, there is an urgent need for the discovery of more effective, selective, more affordable and less toxic treatments.

**Figure 1:**
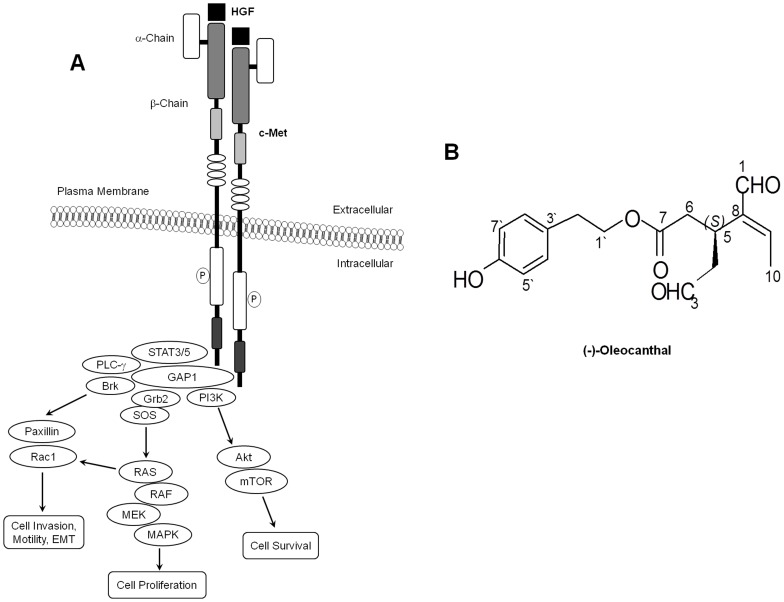
(-)-Oleocanthal and c-Met signaling. (**A**) Schematic representation of HGF/c-Met signaling. (**B**) Chemical structure of (-)-oleocanthal.

The c-Met proto-oncogene encodes a heterodimeric receptor tyrosine kinase (RTK) that consists of an extracellular α-chain and a transmembrane β-chain (Figure 1A) [2], [3]. Hepatocyte growth factor (HGF) binds to the extracellular domain of c-Met with high affinity and induces receptor dimerization with consecutive triggering of c-Met tyrosine kinase activity [4]. This is followed by recruitment and phosphorylation of multiple adaptor proteins as well as activation of signaling molecules such as phosphoinositide-3-kinase (PI_3_K)/Akt, mitogen-activated protein kinase (MAPK), breast tumor kinase (Brk) and phospholipase C-γ (PLC- γ) pathways [4]–[7]. Akt, MAPK and Brk are necessary not only for c-Met-mediated regulation of cell motility, adhesion, and invasion, but also for control of cell survival and mitogenesis [5], [6], [8]. Currently, there is a mounting evidence for the involvement of chronic or dysregulated activation of c-Met receptor tyrosine kinase and its ligand HGF in multiple types of tumor cells leading to enhancing cell growth, angiogenesis, and survival. In addition, aberrant activation of the HGF/c-Met axis is known to promote cytoskeletal changes of many cancer cells in favor for migration, invasion, and eventual metastasis. Therefore, targeting c-Met activity with small molecule inhibitors of the HGF/c-Met axis can be considered a promising approach for cancer treatment and prevention [4]–[6], [8].

It is suggested that the incidence of breast cancer in Mediterranean countries is lower than in the US. This may be partly attributed to the Mediterranean dietary regimens traditionally known to be rich in extra-virgin olive oil (EVOO) [9]. (-)-Oleocanthal (Figure 1B) is a naturally occurring secoiridoid from EVOO, which showed potent anti-inflammatory and neuroprotective activities [10], [11]. In the past few years, there has been an increasing interest in the biological effects of (-)-oleocanthal in inflammation, Alzheimer’s disease and cancer [10]–[21]. In addition, (-)-oleocanthal treatment inhibited the proliferation, migration, and invasion of various human breast, prostate cancer and multiple myeloma cells [12], [13], [17]. Moreover, it showed anti-angiogenic activity by downregulating the expression of the microvessel density marker CD31 in endothelial colony forming cells [17]. A computer-assisted study identified (-)-oleocanthal as a potential c-Met inhibitor hit [17] which inhibited the activation of c-Met kinase in cell-free Z′-LYTE assay [12], [17], however, the exact antiproliferative, antimigratory, and pro-apoptotic mechanisms of (-)-oleocanthal are not well understood. Therefore, the goal of the current study was to characterize the intracellular mechanisms involved in mediating the anticancer effects of (-)-oleocanthal treatment and the potential involvement of c-Met receptor signaling components in breast cancer.

## Materials and Methods

### Chemicals, Reagents, and Antibodies

All materials were purchased from Sigma-Aldrich (St. Louis, MO), unless otherwise stated. (-)-Oleocanthal was isolated from extra-virgin olive oil (Daily Chef, batch number: L022RE-565, Italy). Z-VAD-FMK was purchased from Santa Cruz Biotechnology (Santa Cruz, CA). All antibodies were purchased from Cell Signaling Technology (Beverly, MA), unless otherwise stated. Antibody for Brk was obtained from Abnova (Walnut, CA). Antibody for p-Brk was purchased from Santa Cruz Biotechnology (Santa Cruz, CA). Goat anti-rabbit and goat anti-mouse secondary antibodies were purchased from PerkinElmer Biosciences (Boston, MA). HGF and epidermal growth factor (EGF) were purchased from PeproTech Inc., (Rocky Hill, NJ).

### Cell Lines and Culture Conditions

The human breast cancer cell lines MDA-MB-231, MCF-7 and BT-474 were purchased from ATCC. The human breast cancer cell line MDA-MB-231/GFP was purchased from Cell Biolabs, Inc. (San Diego, CA). The cell lines were maintained in RPMI-1640 supplemented with 10% fetal bovine serum (FBS), 100 U/ml penicillin, 0.1 mg/ml streptomycin in a humidified atmosphere of 5% CO_2_ at 37°C. The MCF10A cell line, an immortalized, non-tumorigenic human mammary epithelial cell line, was purchased from ATCC. MCF10A cells were maintained in DMEM/F12 supplemented with 5% horse serum, 0.5 µg/ml hydrocortisone, 20 ng/ml EGF, 100 U/ml penicillin, 0.1 mg/ml streptomycin, and 10 µg/ml insulin. All cells were maintained at 37°C in an environment of 95% air and 5% CO_2_ in humidified incubator. (-)-Oleocanthal and SU11274 were first dissolved in a volume of DMSO to provide a final 25 mM stock solution. These stock solutions were then used to prepare various concentrations of treatment media. Final concentration of DMSO was maintained as the same in all treatment groups within a given experiment and never exceeded 0.1%. SU11274 is a selective c-Met inhibitor which exhibits greater than 50-fold selectivity for c-Met versus Flk and more than 500 times selectivity versus other tyrosine kinases such as FGFR-1, c-Src, PDGFβR, and EGFR [22]. SU11274 inhibits cell viability and migration in c-Met-expressing cancer cells and abrogates HGF-induced phosphorylation of c-Met and its downstream signaling [22]. SU11274 was used in the experiments as a positive control. The 10 µM dose of SU11274 was used in the assays based on earlier antiproliferative studies (data not shown).

### Measurement of Viable Cell Number

Viable cell count was determined using the 3-(4,5-dimethylthiazol-2yl)-2,5-diphenyl tetrazolium bromide (MTT) colorimetric assay. The optical density of each sample was measured at 570 nm on a microplate reader (BioTek, VT). The number of cells/well was calculated against a standard curve prepared by plating various concentrations of cells, as determined using a hemocytometer at the start of each experiment.

### Cell Growth and Viability Studies

To optimize the concentration of HGF which induces maximum growth of human breast cancer cell lines after 72 h treatment period, MDA-MB-231, MCF-7 or BT-474 cells were plated at a density of 1×10^4^ cells per well (6 wells/group) in 96-well culture plates and maintained in RPMI-1640 media supplemented with 10% FBS and allowed to adhere overnight. The next day, cells were washed with phosphate buffer saline (PBS), divided into different treatment groups and then given various concentrations of HGF in serum-free media. Cells in all groups were fed fresh treatment media every other day for a 72 h treatment period. Viable cell number was determined every day using the MTT assay.

To evaluate the effect of (-)-oleocanthal on the proliferation and growth of breast cancer cell lines, growth studies were performed. For growth studies, MDA-MB-231, MCF-7 or BT-474 cells were plated at a density of 1×10^4^ cells per well (6 wells/group) in 96-well culture plates and maintained in RPMI-1640 media supplemented with 10% FBS and allowed to adhere overnight. The next day, cells were washed with PBS, divided into different treatment groups and then given various concentrations of (-)-oleocanthal in serum-free media containing 40 ng/ml HGF (which induced maximum growth in the three cell lines after 72 h) or no HGF (0.5% FBS was added to the media to maintain the viability of the cells throughout the experiment). Cells in all groups were fed fresh treatment media every other day for a 72 h treatment period. Viable cell number was determined every day using the MTT assay.

To evaluate the effect of (-)-oleocanthal on the proliferation and growth of immortalized non-tumorigenic mammary cells, MCF10A cells were plated at a density of 1×10^4^ cells per well (6 wells/group) in 96-well culture plates and maintained in DMEM/F12 media containing 5% horse serum and allowed to attach overnight. The next day, cells were washed with PBS, divided into different treatment groups and then given various concentrations of (-)-oleocanthal in serum-free defined media containing 40 ng/ml HGF. Cells in all groups were fed fresh treatment media every other day for a 72 h treatment period. Viable cell number was determined every day using the MTT assay.

### Western Blot Analysis

To study treatment effects of (-)-oleocanthal on MDA-MB-231 cell cycle progression, cells in the various treatment groups were synchronized in G1 phase [23]. Briefly, MDA-MB-231 cells were plated at a density of 1×10^6^ cells/100 mm culture plates in RPMI-1640 media supplemented with 10% FBS and allowed to adhere overnight. Cells were then washed twice with PBS and starved in control or treatment serum-free medium containing 0.5% FBS for 48 h to synchronize the cells in G1 phase. Afterwards, cells were fed various doses of (-)-oleocanthal in serum-free defined media containing 40 ng/ml HGF as the mitogen for 24 h. To study the effect of (-)-oleocanthal treatment on c-Met, Akt, and MAPK phosphorylation, MDA-MB-231 cells were plated at a density of 1×10^6^ cells/100 mm culture plates in RPMI-1640 media supplemented with 10% FBS and allowed to adhere overnight. Cells were then washed twice with PBS and starved in control or treatment medium containing 0.5% FBS for 72 h and stimulated with 100 ng/ml human recombinant HGF for 10 min before cell lysis. In order to examine whether or not caspase-3 and caspase-8 activation were involved in the apoptosis triggered by (-)-oleocanthal, MDA-MB-231 cells were plated at a density of 3×10^6^ cells/100 mm culture plates, and then allowed to attach overnight. Cells were then washed with PBS and pretreated with or without caspase family inhibitor Z-VAD-FMK (50 µM) for 3 h. Cells were then further incubated with DMSO (control) or (-)-oleocanthal (25 µM) for another 24 h in serum-free defined media containing 40 ng/ml HGF. In all other Western blot experiments, cells were plated at a density of 1×10^6^ cells/100 mm culture plates, allowed to attach overnight and then washed with PBS and incubated in the respective control or treatment in serum-free defined media containing 40 ng/ml HGF as the mitogen for 72 h. In case of *in vivo* experiment, breast tumor tissues were stored at −80°C until protein extraction. At the end of treatment period, cells were lysed in RIPA buffer (Qiagen Sciences Inc., Valencia, CA) and breast tumor tissues were homogenized in RIPA buffer using an electric homogenizer. Protein concentration was determined by the BCA assay (Bio-Rad Laboratories, Hercules, CA). Equivalent amounts of protein were electrophoresed on SDS–polyacrylamide gels. The gels were then electroblotted onto PVDF membranes. These PVDF membranes were then blocked with 2% BSA in 10 mM Tris-HCl containing 50 mM NaCl and 0.1% Tween 20, pH 7.4 (TBST) and then, incubated with specific primary antibodies overnight at 4°C. At the end of incubation period, membranes were washed 5 times with TBST and then incubated with respective horseradish peroxide-conjugated secondary antibody in 2% BSA in TBST for 1 h at room temperature followed by rinsing with TBST for 5 times. Blots were then visualized by chemiluminescence according to the manufacturer’s instructions (Pierce, Rockford, IL). Images of protein bands from all treatment groups within a given experiment and scanning densitometric analysis were acquired using Kodak Gel Logic 1500 Imaging System (Carestream Health Inc, New Haven, CT). The visualization of β-tubulin was used to ensure equal sample loading in each lane. All experiments were repeated at least 3 times.

### Analysis of Cell Cycle Progression by Flow Cytometry

To study treatment effects on cell cycle, MDA-MB-231 cells were plated and then synchronized in G1 phase and fed treatments as described above. At the end of the experiment, cells in the various treatment groups were isolated with trypsin and then resuspended in ice cold PBS, fixed with cold (−20°C) 70% ethanol, and stored at 4°C for 2 h. Afterwards, cells were rehydrated with ice cold PBS and then incubated with DNA staining buffer (sodium citrate 1 mg/ml, triton-X 100 3 µl/ml, propidium iodide 100 µg/ml, ribonuclease A 20 µg/ml) for 30 min at 4°C in the dark. DNA content was then analyzed using a FACS Calibur flow cytometer (BD Biosciences, San Jose, CA). For each sample, 10,000 events were recorded, and histograms were generated using CellQuest software (BD Biosciences, San Jose, CA). All experiments were repeated at least three times.

### Wound-healing Assay

The *in vitro* wound-healing assay was used to assess directional cell motility in two dimensions. MDA-MB-231 cells were plated in sterile flat-bottom 24-well plates (6 replicates/group) and allowed to form a subconfluent cell monolayer per well overnight. Wounds were then scratched in each cell monolayer using a sterile 200 µl pipette tip. Media was removed and cells were washed twice with PBS and once with fresh serum-free media to remove floating cells. Cells were then incubated in culture media containing (-)-oleocanthal or SU11274 at the desired concentrations in serum-free defined media containing 40 ng/ml HGF as the mitogen. Cell were incubated for a 24 h culture period and afterward, media was removed and cells were washed with pre-cooled PBS, fixed with methanol previously cooled to −20°C, and stained with Giemsa. Wound healing was visualized at 0 and 24 h by Nikon ECLIPSE TE200-U microscope (Nikon Instruments Inc., Melville, NY). Digital images were captured using Nikon NIS Elements software (Nikon Instruments Inc., Melville, NY). The distance traveled by the cells was determined by measuring the wound width at time 24 h and subtracting it from the wound width at the start of treatment (time zero). The values obtained were then expressed as % migration, setting the gap width at *t_0_* as 100%. Each experiment was done in triplicate and the distance migrated was calculated in three or more randomly selected fields per treatment group.

### Cell Invasion Assay

MDA-MB-231 cell invasion was determined using the CytoSelect Cell Invasion Assay (Cell Biolabs, Inc., San Diego, CA) according to manufacturer’s instructions. Briefly, MDA-MB-231 cells were pre-treated with (-)-oleocanthal for 24 h. Basement membranes of Boyden chambers were rehydrated with 300 µl serum free RPMI-1640, and 3×10^5^ cells were then seeded into the upper area of the chamber in serum free RPMI-1640. Bottom wells were filled with defined control serum-free media supplemented with 40 ng/ml HGF containing (-)-oleocanthal or no (-)-oleocanthal. After 24 h incubation (37°C, 5% CO_2_), non-invasive cells were removed from the upper chamber and cell invasion was assessed by light microscopy after staining of invaded cells with crystal violet Cell Stain Solution (Cell Biolabs, CA). For colorimetric quantification of invasion, inserts were then placed in extraction buffer (200 µl, 10 min), and absorbance at 560 nm was determined after transfer to a 96 well plate (100 µl per well) using a BioTek microtiter plate reader (BioTek, VT).

### Apoptosis Analysis with Annexin V Staining by Flow Cytometry

Induction of apoptosis was assessed by the binding of annexin V to phosphatidylserine, which is externalized to the outer leaflet of plasma membrane early during induction of apoptosis. Analysis of annexin V was determined using Annexin V-FITC Early Apoptosis Detection Kit (Cell Signaling Technology, Beverly, MA). Cells were plated at a density of 5×10^6^ cells/100 mm culture plates, allowed to attach overnight. Afterwards, cells were incubated in the respective control or (-)-oleocanthal treated defined serum-free medium containing 40 ng/ml of HGF for 24 h. At the end of the experiment, cells in each treatment group were isolated with trypsin and then washed twice with ice cold PBS. Cells were then resuspended in 96 µl of ice-cold 1X Annexin V Binding Buffer. Afterwards, 1 µl Annexin V-FITC Conjugate and 12.5 µl Propidium Iodide (PI) Solution were added to each 96 µl cell suspension. The cells were then incubated for 10 min on ice in the dark. The cell suspension was then diluted to a final volume of 250 µl per assay with ice-cold, 1X Annexin V Binding Buffer. Dot plots were generated using CellQuest software (BD Biosciences, San Jose, CA), and they were divided into 4 quadrants (LL: lower left; LR: lower right; UL: upper left; UR: upper right). The LL quadrant shows cells negative for both annexin V and PI (living, non-apoptotic cells). The LR quadrant shows cells positive for annexin V, but negative for PI (living, early apoptotic). The UL quadrant shows cells positive for PI, but negative to annexin V (dead), whereas the UR quadrant shows cells positive for both annexin V and PI (late apoptotic). All experiments were repeated at least three times.

### RNA Interference

Transfection of small interfering RNA (siRNA) into cells was conducted when the cells reached 70% confluence. The siRNAs of c-Met and a non-targeting control were purchased from Cell Signaling Technology (Beverly, MA). Experiments were conducted using Lipofectamine RNAiMAX Reagent (Carlsbad, CA) as a transfection agent and siRNA, and experiments were conducted according to the manufacturers’ instructions.

### Xenograft Studies

All animal experiments were approved by the Institutional Animal Care and Use Committee, University of Louisiana at Monroe, and were handled in strict accordance with good animal practice as defined by the NIH guidelines. Athymic nude mice (Foxn1^nu^/Foxn1^+^, 4–5 weeks, female) were purchased from Harlan (Indianapolis, IN). The mice had free access to standard pellet food and water. The animals were acclimated to animal house facility conditions at a temperature of 18–25°C, with a relative humidity of 55 to 65% and a 12 h light/dark cycle, for one week prior to the experiments. MDA-MB-231/GFP human breast cancer cells were cultured and resuspended in serum-free DMEM medium (20 µl). After anesthesia, cell suspensions (1×10^6^ cells/20 µl) were inoculated subcutaneously into the second mammary gland fat pad just beneath the nipple of each animal to generate orthotopic breast tumors. At 48 h post-inoculation, the mice were randomly divided into two groups: i) the vehicle-treated control group (n = 5), ii) the (-)-oleocanthal-treated group (n = 5). Treatment (3X/week) started 5 days postinoculation with intraperitoneal (i.p.) administered vehicle control (DMSO/saline) or 5 mg/kg (-)-oleocanthal. Selection of this dose was based on earlier *in vivo* studies on oleocanthal [11]. (-)-Oleocanthal treatment was prepared by dissolving 5 mg of (-)-oleocanthal in 100 µl DMSO to prepare a stock solution, then dissolving 20 µl of stock solution in 980 µl normal saline just prior to the injection. The mice were monitored by measuring tumor volume, body weight, and clinical observation. Tumor volume (V) was calculated by V = L/2 x W^2^, where L was the length and W was the width of tumors. All the mice were sacrificed at day 33 postinoculation, and the tumors were excised and weighed. Breast tumor tissues were stored at −80°C until total protein extraction for Western blot analysis.

### Immunohistochemistry

The tumor specimens were processed with the use of alcohols and xylene and then infiltrated in paraffin wax using the Excelsior ES Tissue Processor. Paraffin sections were dewaxed in xylene, rinsed in grade alcohol, and rehydrated in water and then were placed in citric buffer (PH 6.0) and treated in a microwave oven with high power for 3 min and 10% goat serum for 30 min. Subsequently, antibodies with proper dilution were applied on the sections as follows: CD31 (Pierce Product# PA5-32321; 1∶50 dilution, 1 h at RT) and Ki-67 (Cell Signaling Product# #9027; 1∶150 dilution, 1 h at RT). Following that, secondary antibodies (Ventana Multimer Anti Rb-HRP Product#760-4311 24 min at RT) were applied. Signals were developed with Vector ImmPACT DAB Product#SK-4105 for 8 mins at RT. The sections were finally counter stained by hematoxylin solution for 1 min at RT.

### Determination of Positive Ki-67 Cells and Microvessel Density

To evaluate positive Ki-67 cells in breast cancer tissues, 5 areas were examined at a magnification of ×200. Microvessel density (MVD) of breast tumor tissue sections was evaluated. Any CD31+stained endothelial cell or endothelial cell cluster was counted as one microvessel. The mean microvessel count of the five most vascular areas was taken as the MVD, which was expressed as the absolute number of microvessels per 1.485 mm^2^ (×200 field).

### Statistics

The results are presented as means ± SEM of at least three independent experiments. Differences among various treatment groups were determined by the analysis of variance (ANOVA) followed by Dunnett’s test using PASW statistics version 18. A difference of *P*<0.05 was considered statistically significant as compared to the vehicle-treated control group. The IC_50_ values (concentrations that induce 50% cell growth inhibition) were determined using non-linear regression curve fit analysis using GraphPad Prism software version 5.

## Results

### Effect of (-)-oleocanthal on Breast Cancer Cell Growth

**Figure 2:**
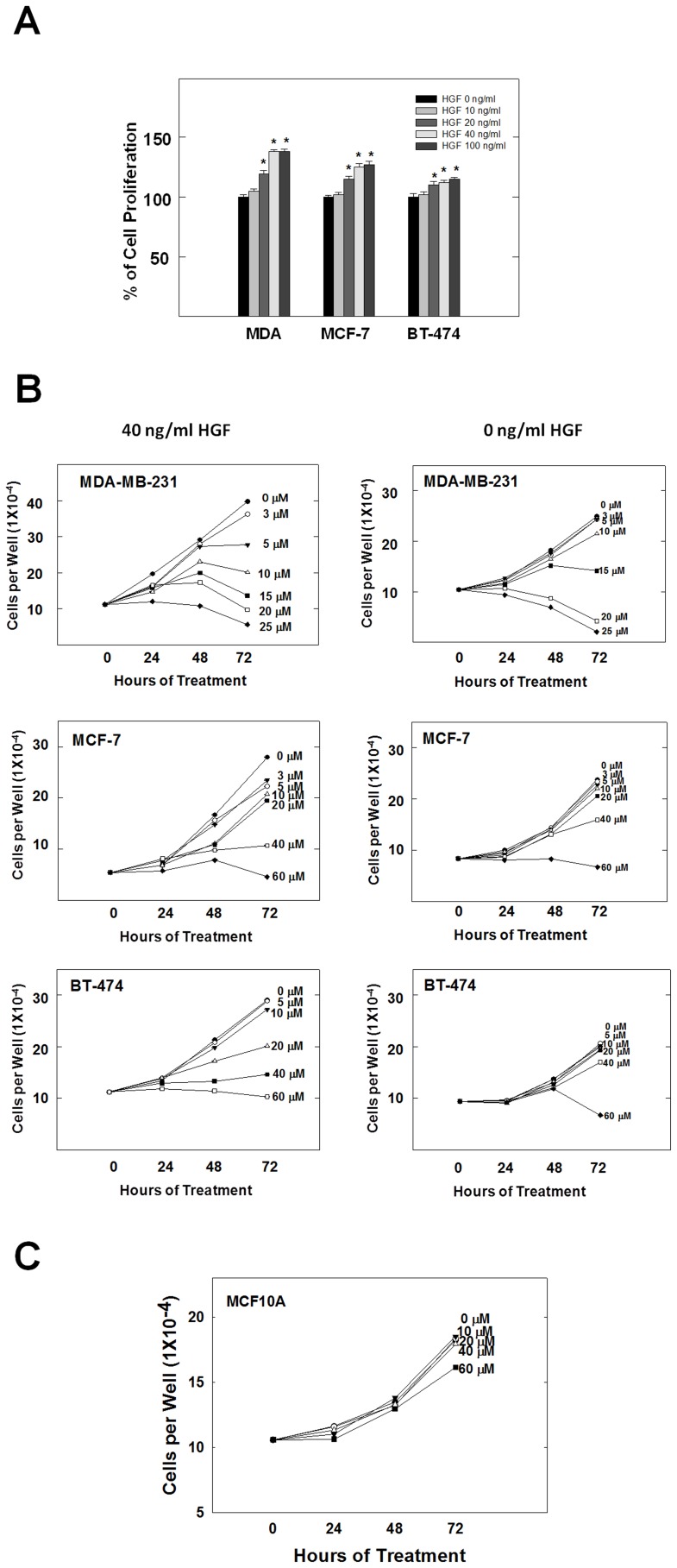
(-)-Oleocanthal inhibits HGF-induced proliferation of MDA-MB-231, MCF-7 and BT-474 breast cancer cell lines. (**A**) HGF stimulates the growth of human breast cancer cells in a dose-dependent manner with maximum effect at 40 ng/ml after 72 h culture period. (**B**) Effects of (-)-oleocanthal treatment on growth of MDA-MB-231, MCF-7 and BT-474 cancer cells in the presence or absence of 40 ng/ml HGF after 72 h treatment period. (**C**) Effects of (-)-oleocanthal treatment on the viability of non-tumorigenic human MCF10A mammary epithelial cells after a 72 h treatment period. In these assays, cells were plated at a density of 1×10^4^ cells per well in 96-well plates and maintained in media supplemented with 10% FBS and allowed to adhere overnight. The next day, cells were washed with PBS, divided into different treatment groups. Cells were fed fresh treatment media every other day for a 72 h treatment period. Viable cell count was determined by MTT assay. Vertical bars indicate the mean cell count ± SEM in each treatment group. *P<0.05 as compared with vehicle-treated controls.

The activation of c-Met with HGF is known to play an important role in cell proliferation in many kinds of cancer cells. Therefore, the role of HGF on the proliferation and growth of MDA-MB-231, MCF-7 and BT-474 breast cancer cells was investigated (Figure 2A). Cell proliferation assay was performed in cells treated with HGF at concentrations of 0, 10, 20, 40, and 100 ng/ml. MTT assay results after 72 h treatment showed that HGF caused a dose-dependent increase in breast cancer cells proliferation (Figure 2A) and the maximum effect was identified at 40 ng/ml of HGF (comparable to 100 ng/ml) in all three breast cancer cell lines (Figure 2A).

The antiproliferative effects of various doses of (-)-oleocanthal on HGF-mediated growth of MDA-MB-231, MCF-7 and BT-474 breast cancer cells after 24, 48 and 72 h culture periods are shown in Figure 2B. HGF at a concentration of 40 ng/ml was used in growth studies as it showed maximum growth induction in the three human breast cancer cell lines investigated. (-)-Oleocanthal treatment caused a dose-dependent suppression of HGF-induced proliferation of MDA-MB-231, MCF-7 and BT-474 cells after 48 and 72 h (Figure 2B, left panel). However, larger concentrations of (-)-oleocanthal were required to significantly abolish cell viability of MDA-MB-231, MCF-7 and BT-474 cells grown in HGF-free media after 72 h with a little or no effect after 48 h (Figure 2B, right panel). The IC_50_ values for (-)-oleocanthal treatment in HGF-supplemented media were 10.9, 20.1 and 25.4 µM in MDA-MB-231, MCF-7 and BT-474 breast cancer cells, respectively. However, the IC_50_ values for (-)-oleocanthal treatment in HGF-free media were 16.2, 40.8 and 58.4 µM in MDA-MB-231, MCF-7 and BT-474 breast cancer cells, respectively. These results indicate that (-)-oleocanthal treatment inhibits HGF-dependent MDA-MB-231, MCF-7 and BT-474 cell growth in a dose and time-responsive manner as compared to cells in the vehicle-treated control groups (Figure 2B).

### Effects of (-)-oleocanthal on Non-tumorigenic Human Mammary Epithelial Cell Growth

Treatment effects of (-)-oleocanthal on the growth of immortalized non-tumorigenic human (MCF10A) mammary epithelial cells over 24, 48 and 72 h culture periods are shown in Figure 2C. Results showed that treatment with 0–40 µM (-)-oleocanthal had no effect on MCF10A cell viability as compared to their respective vehicle treated control groups (Figure 2C). In contrast, treatment with 60 µM caused a significant cell growth inhibition only after 72 h incubation period. These results suggest selectivity of (-)-oleocanthal antiproliferative effect toward breast cancer cells.

### Effects of (-)-oleocanthal Treatment on Cell Cycle Progression and HGF-stimulated Akt and MAPK Phosphorylation

**Figure 3:**
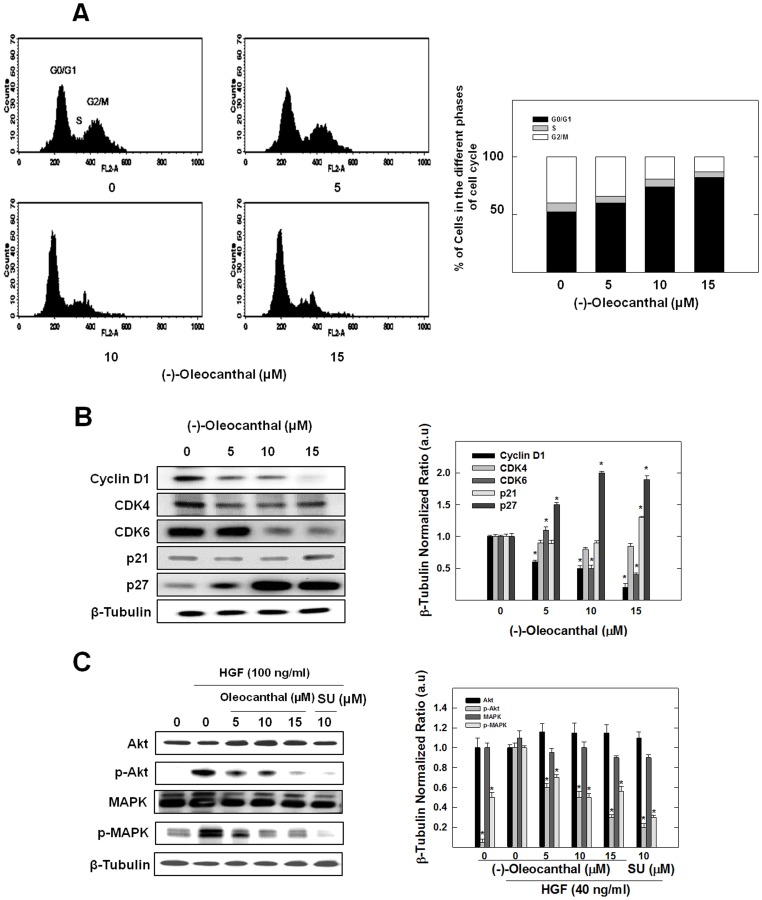
(-)-Oleocanthal treatment inhibits HGF-induced G1/S cell cycle progression and mitogenic signaling in human MDA-MB-231 breast cancer cells. (**A**) Flow cytometry analysis for cell cycle progression in control and (-)-oleocanthal-treated MDA-MB-231 cells. Cells in the various treatment groups were synchronized in G1 phase. Briefly, MDA-MB-231 cells were plated at a density of 1×10^6^ cells/100 mm plates in RPMI-1640 media supplemented with 10% FBS and allowed to adhere overnight. Cells were then washed twice with PBS and starved in control or treatment serum-free medium containing 0.5% FBS for 48 h to synchronize the cells in G1 phase. Afterwards, cells were fed various doses of (-)-oleocanthal in serum-free defined media containing 40 ng/ml HGF as the mitogen for 24 h. *Left panel* shows histograms generated using CellQuest software (PI staining). *Right panel* shows percentage of cells in each phase of cell cycle. Vertical bars show the average of 3 independent experiments. (**B**) Western blot analysis showing (-)-oleocanthal treatment effects on G1/S cell cycle regulatory proteins. Cells in the various treatment groups were synchronized in G1 phase in the same way described above. (-)-Oleocanthal treatment caused a marked downregulation of cyclin D1 and CDK6, while it caused upregulation of p21 and p27. (**C**) Western blot analysis showing (-)-oleocanthal treatment effects on c-Met downstream mitogenic signaling proteins Akt and MAPK. MDA-MB-231 cells were plated at a density of 1×10^6^ cells/100 mm culture plates in RPMI-1640 media supplemented with 10% FBS and allowed to adhere overnight. Cells were then washed twice with PBS and starved in control or treatment medium containing 0.5% FBS for 72 h and stimulated with 100 ng/ml human recombinant HGF for 10 min before cell lysis. SU11274 was used as a positive control. Afterwards, whole cell lysates were prepared for subsequent separation by polyacrylamide gel electrophoresis followed by Western blot analysis. Scanning densitometric analysis was performed on all blots done in triplicate and the integrated optical density of each band was normalized with corresponding β-tubulin, as shown in bar graphs beside their respective Western blot images. Vertical bars in the graph indicate the normalized integrated optical density of bands visualized in each lane ± SEM, *P<0.05 as compared with vehicle-treated controls.

The effects of (-)-oleocanthal treatment on cell cycle progression was evaluated using flow cytometry and Western blot analysis (Figures 3A and B). MDA-MB-231 cells exposed to various concentrations of (-)-oleocanthal resulted in a dose-dependent increase in the proportion of cells in G1 phase of the cell cycle from 50% (vehicle-treated control) to nearly 82% with 15 µM (-)-oleocanthal treatment (Figure 3A). These studies also showed that no sub-G1 population of cells was observed in any of the treatment groups (0–15 µM), indicating that none of the treatments initiated apoptosis (programmed cell death) in MDA-MB-231 cells at these concentrations.

Additional studies were conducted to determine the effects of (-)-oleocanthal treatment on the relative intracellular levels of cyclins, cyclin-dependent kinases (CDKs), cyclin-dependent kinase inhibitors (CKIs) and mitogenic signaling proteins as determined by Western blot analysis (Figures 3B and C). Treatment with (-)-oleocanthal resulted in a prominent reduction in cyclin D1 levels as compared to the vehicle-treated control group (Figure 3B). In addition, treatment of MDA-MB-231 cells with 5–15 µM (-)-oleocanthal was not found to have any effect on the relative levels of CDK4 (Figure 3B). However, (-)-oleocanthal treatment caused a relatively large reduction in CDK6 levels and marked increase in the levels of the CKI proteins p21 and p27, compared to the vehicle-treated controls (Figure 3B).

Results also showed that (-)-oleocanthal treatment caused a dose-dependent inhibition of HGF-induced Akt and MAPK phosphorylation, important downstream signaling molecules in c-Met signaling. This effect was comparable to the known c-Met inhibitor SU11274 (Figure 3C).

### Effects of (-)-oleocanthal on HGF-induced Mammary Tumor Cell Migration and Invasion and its Associated Brk/paxillin/Rac1 Signaling

**Figure 4:**
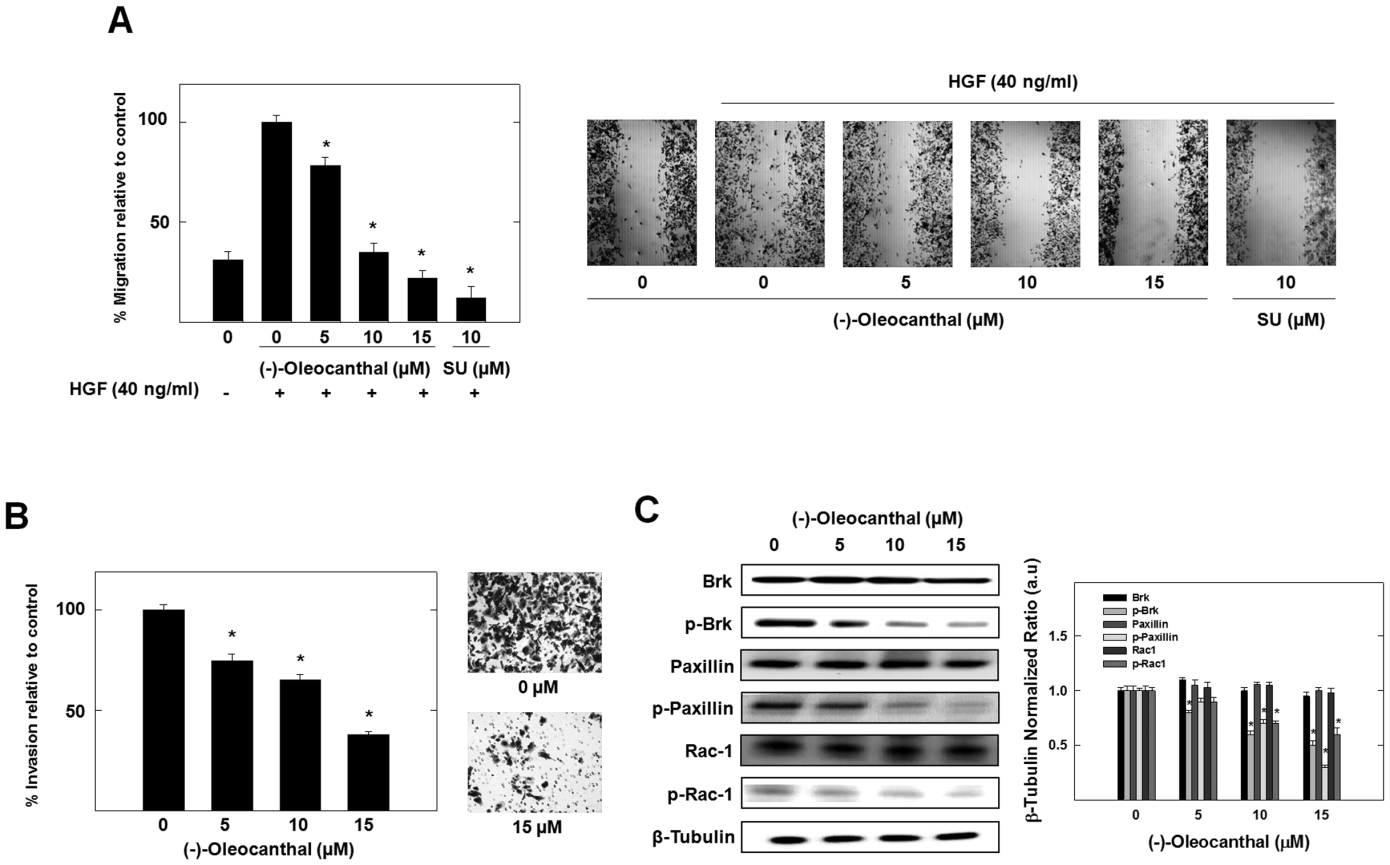
(-)-Oleocanthal treatment caused a dose-dependent suppression of HGF-induced mammary tumor cell migration and invasion and Brk/paxillin/Rac1 pathway signaling. (**A**) Wound healing assay. *Left panel* shows quantitative analysis of the percentage of gap reduction (i.e., wound closure) in various treatment groups in MDA-MB-231 cancer cells. Vertical bars indicate the percentage of wound closure at 24 h after wounding was calculated relative to the wound distance at time 0 (*t_0_*) ± SEM in each treatment group. *P<0.05 as compared with vehicle-treated control. *Right panel* represents photomicrographs of wound healing assay showing (-)-oleocanthal treatment blocked the migration MDA-MB-231 cells in response to HGF stimulation. The treatment with 10 µM SU11274 was used as a positive control. (**B**) Transwell invasion chamber assay. The cells were treated with 5, 10, and 15 µM (-)-oleocanthal for 24 h. *Left panel* shows quantitative analysis of the percentage of cells invading the basement membrane at the end of treatment period. Vertical bars indicate the percentage of cells invading the basement membrane ± SEM in each treatment group. *P<0.05 as compared with vehicle-treated controls. *Right panel* represents photomicrographs of cells invading the basement membrane and 15 µM (-)-oleocanthal treatment blocked the invasion of MDA-MB-231 cells. (**C**) Western blot analysis showing (-)-oleocanthal treatment effects on Brk/Paxillin/Rac1 pathway signaling after 72 h treatment in MDA-MB-231 cancer cells. Cells were plated at 1×10^6^ cells/100 mm culture plates, allowed to attach overnight and then washed with PBS and incubated in the respective control or treatment in serum-free defined media containing 40 ng/ml HGF as the mitogen for 72 h. Afterwards, whole cell lysates were prepared for subsequent separation by polyacrylamide gel electrophoresis followed by Western blot analysis. Scanning densitometric analysis was performed on all blots done in triplicate and the integrated optical density of each band was normalized with corresponding β-tubulin, as shown in bar graphs beside their respective Western blot images. Vertical bars in the graph indicate the normalized integrated optical density of bands visualized in each lane ± SEM, *P<0.05 as compared with vehicle-treated controls.

The important characteristic of metastasis is the migratory and invasive ability of tumor cells. To test the effect of (-)-oleocanthal on HGF-induced MDA-MB-231 cell migration, wound healing assay was performed (Figure 4A). HGF at 40 ng/ml induced cellular migration with more than 85% wound closure after a 24 h treatment period. Figure 4A shows the ability of (-)-oleocanthal to significantly suppress HGF-induced cell migration in a dose-dependent manner. Treatment of the cells with 5, 10, and 15 µM (-)-oleocanthal for 24 h inhibited cell migration by 22%, 65%, and 78%, respectively (Figure 4A). A 10 µM treatment with the known c-Met kinase inhibitor SU11274 inhibited cell migration by 88%. The effect of (-)-oleocanthal on cell invasion was examined using transwell chamber assay. As shown in Figure 4B, (-)-oleocanthal significantly decreased the level of HGF-mediated cell invasion through the matrigel in a dose-dependent manner. Treatment of MDA-MB-231 breast cancer cells with 5, 10, and 15 µM (-)-oleocanthal for 24 h inhibited the number of cells invading the lower chamber by 26%, 35%, and 62%, respectively (Figure 4B).

To study the effects of (-)-oleocanthal treatment on Brk/Paxillin/Rac1 pathway, Western blot analysis was performed (Figure 4C). Results showed potent dose-dependent inhibition of Brk phosphorylation after treatment with (-)-oleocanthal for 72 h in MDA-MB-231 cancer cells compared to the vehicle-treated control group. Alternatively, (-)-oleocanthal treatment had no effects on the total levels of Brk in treated cells. Moreover, the effect of (-)-oleocanthal on Brk phosphorylation was associated with dose-dependent suppression of paxillin and Rac1 phosphorylation without affecting their total levels. These results suggest that (-)-oleocanthal significantly blocked HGF-induced migration and invasion of the highly invasive MDA-MB-231 breast cancer cells. This effect might be related, at least in part, to the suppression of Brk/paxillin/Rac1 signaling pathway.

### Effect of (-)-oleocanthal on HGF-induced c-Met Phosphorylation and Epithelial-to-mesenchymal Transition (EMT)

**Figure 5:**
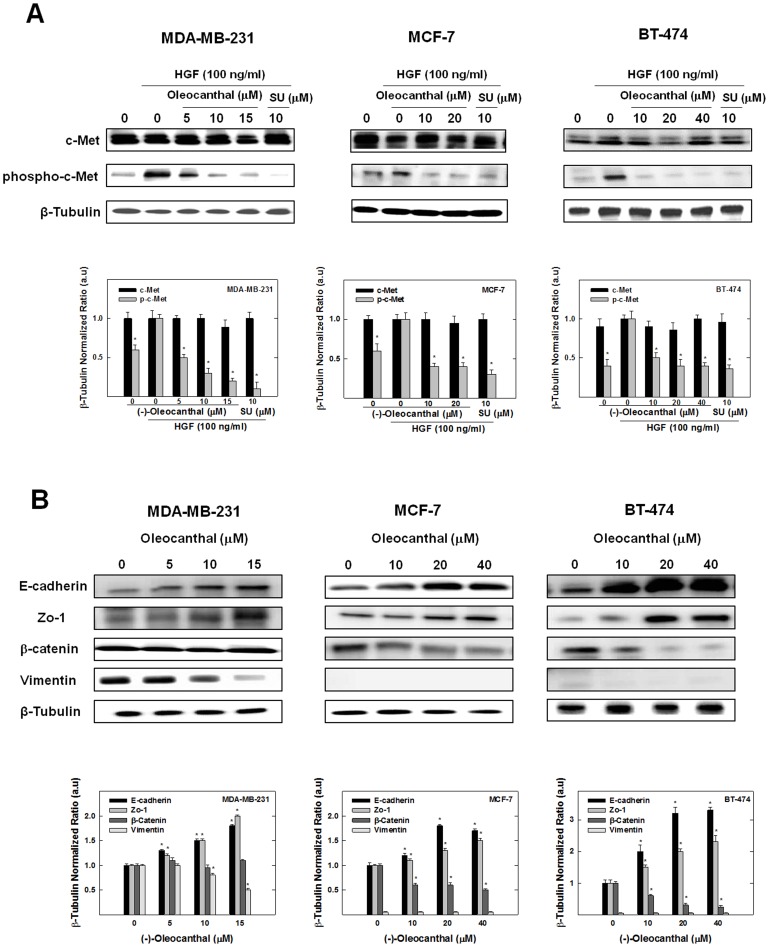
(-)-Oleocanthal treatment inhibits HGF-induced Met activation, stabilizes the epithelial phenotype, and reduces mesenchymal phenotype in breast cancer cells. (**A**) Western blot analysis. (-)-Oleocanthal caused a dose-dependent inhibition of HGF-induced phosphorylation of c-Met in MDA-MB-231, MCF-7, and BT-474 breast tumor cells with no effect on total Met levels. Cells were plated at 1×10^6^ cells/100 mm culture plates in RPMI-1640 media supplemented with 10% FBS and allowed to adhere overnight. Cells were then washed twice with PBS and starved in control or treatment medium containing 0.5% FBS for 72 h and stimulated with 100 ng/ml human recombinant HGF for 10 min before cell lysis. SU11274 was used as a positive control. (**B**) Western blot analysis. (-)-Oleocanthal resulted in a marked increase in the level of epithelial markers E-cadherin and Zo-1 in MDA-MB-231, MCF-7, and BT-474 cells and a decrease of mesenchymal marker vimentin expression (in MDA-MB-231 cells) compared to the vehicle-treated control groups. (-)-Oleocanthal treatment resulted in downregulation of β-catenin in MCF-7 and BT-474 cells. Cells were plated at 1×10^6^ cells/100 mm culture plates, allowed to attach overnight and then washed with PBS and incubated in the respective control or treatment in serum-free defined media containing 40 ng/ml HGF as the mitogen for 72 h. Whole cell lysates were prepared for subsequent separation by polyacrylamide gel electrophoresis followed by Western blot analysis. Scanning densitometric analysis was performed on all blots done in triplicate and the integrated optical density of each band was normalized with corresponding β-tubulin, as shown in bar graphs below their respective Western blot images. Vertical bars in the graph indicate the normalized integrated optical density of bands visualized in each lane ± SEM, *P<0.05 as compared with vehicle-treated controls.

In this study, MDA-MB-231, MCF-7, and BT-474 human breast cancer cells were used to assess the effect of (-)-oleocanthal on HGF-induced c-Met phosphorylation (activation) (Figure 5A). Phospho-c-Met refers to the phosphorylation of the kinase domain at Y1234/1235. Western blot analysis results showed that (-)-oleocanthal treatment caused a dose-dependent inhibition of HGF-induced phosphorylation of c-Met in the three breast cancer cell lines investigated. However, (-)-oleocanthal treatment did not affect the total levels of c-Met at the doses used for the treatment of the three breast cancer cell lines (Figure 5A). In addition, treatment of MDA-MB-231, MCF-7, and BT-474 human breast cancer cells with (-)-oleocanthal resulted in a marked increase in the levels of epithelial markers E-cadherin and zona occludens 1 (Zo-1) in the three cell lines, and decreased the expression of the mesenchymal marker vimentin in MDA-MB-231, compared to the vehicle-treated control groups (Figure 5B). However, vimentin was below the level of detection in MCF-7 and BT-474 cells. In addition, (-)-oleocanthal treatment showed cell line-specific change of β-catenin levels. In MDA-MB-231, (-)-oleocanthal treatment resulted in little or no change of β-catenin expression, while it caused a dose-dependent reduction of β-catenin expression levels in MCF-7 and BT-474 cells (Figure 5B). Therefore, it can be concluded that (-)-oleocanthal stabilizes the epithelial phenotype, and reduces mesenchymal phenotype in breast cancer cells.

### Pro-apoptotic Effects of (-)-oleocanthal in Breast Cancer Cells

**Figure 6:**
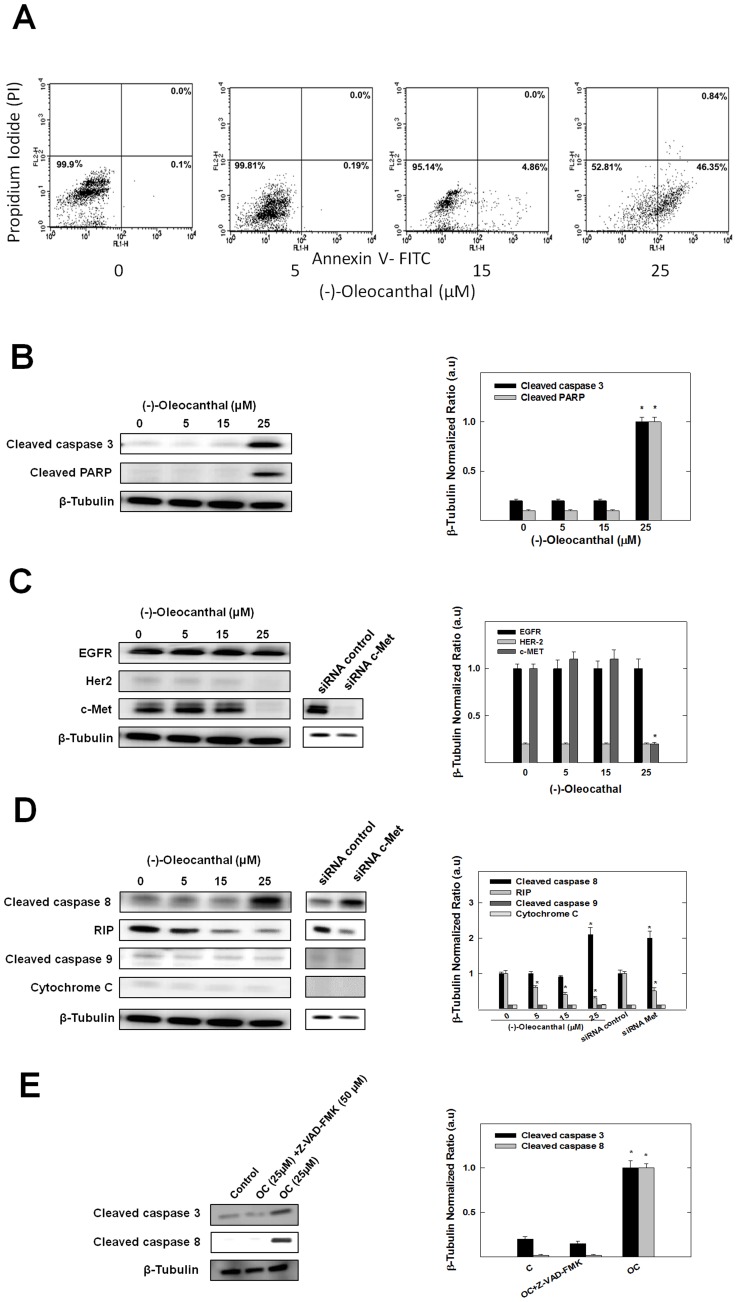
(-)-Oleocanthal treatment induces apoptosis at 25 µM in MDA-MB-231 breast cancer cells. (**A**) Flow cytometry analysis. Cells were plated at a density of 5×10^6^ cells/100 mm culture plates, allowed to attach overnight. Afterwards, cells were incubated in the respective control or (-)-oleocanthal-treated RPMI-1640 medium containing 40 ng/ml HGF for 24 h. At the end of the experiment, cells in each treatment group were trypsinized, washed then resuspended in ice-cold 1X Annexin V Binding Buffer. Afterwards, the cells were treated as described in the Materials and Methods. In the dot plot of double variable flow cytometry, LL quadrant (FITC −/PI -) shows living cells; UR quadrant (FITC +/PI +) stands for late apoptotic cells; and LR quadrant (FITC +/PI -) represents early apoptotic cells. (**B**) Western blot analysis of cleaved caspase 3 and cleaved PARP. (-)-Oleocanthal treatment at 25 µM for 72 h markedly increased levels of cleaved caspase-3 and cleaved PARP. (**C**) Western blot analysis. (-)-Oleocanthal at 25 µM for 72 h downregulates c-Met levels without affecting EGFR levels, and right panel shows that transfection of MDA-MB-231 cells with c-Met-targeted siRNA totally abolished c-Met protein expression. (**D**) Western blot analysis of cleaved caspase 8, RIP, cleaved caspase 9 and cytochrome c. Treatment with 25 µM (-)-oleocanthal increased the cleavage of caspase-8 and RIP, but not caspase-9 or cytochrome c. Right panel shows that c-Met-targeted siRNA yielded a pattern of apoptosis that is similar to that following treatment with (-)-oleocanthal at 25 µM for 72 h by causing an increase in caspase-8 and RIP cleavage, with no effect on caspase-9 and cytochrome c levels. (**E**) Effect of Z-VAD-FMK on (-)-oleocanthal-induced apoptosis. MDA-MB-231 cells were treated with 25 µM (-)-oleocanthal in the presence or absence of caspase inhibitor Z-VAD-FMK (50 µM). After 24-h incubation, cells were analyzed to examine cell death by measuring caspase 3 and caspase 8 cleavage detected by Western blotting. In all the above experiments, whole cell lysates were prepared for subsequent separation by polyacrylamide gel electrophoresis followed by Western blot analysis. Scanning densitometric analysis was performed on all blots done in triplicate and the integrated optical density of each band was normalized with corresponding β-tubulin, as shown in bar graphs beside their respective Western blot images. Vertical bars in the graph indicate the normalized integrated optical density of bands visualized in each lane ± SEM, *P<0.05 as compared with vehicle-treated controls.

The present study has demonstrated that (-)-oleocanthal at 25 µM induced apoptosis in MDA-MB-231 cells. The cells were treated with 5, 15, and 25 µM of (-)-oleocanthal for 24 h. Cell death was assessed after treatment by determination of annexin V (apoptotic marker) and PI (oncotic marker) binding using flow cytometry (Figure 6A). (-)-Oleocanthal produced a concentration-dependent increase in annexin V labeling, in the absence of PI staining, with a maximal increase at 25 µM (46.35% of cells are annexinV-positive) (Figure 6A). Subsequent Western blot analysis showed that (-)-oleocanthal treatment at 25 µM markedly increased levels of cleaved caspase-3 (activated) and cleaved PARP, both of which are positive markers for apoptosis, in these cells following a 72 h culture period (Figure 6B).

EGFR, HER-2, and c-Met are among the most critical proteins for breast cancer proliferation and survival. The effects of (-)-oleocanthal exposure on EGFR, HER-2, and c-Met protein expression were evaluated by Western blotting in MDA-MB-231 cells (Figure 6C). Results revealed that c-Met protein levels were downregulated with (-)-oleocanthal treatment at 25 µM (Figure 6C), while EGFR levels were unchanged (Figure 6C). HER-2 levels were barely detectable in this cell line (MDA-MB-231 cells are classified as triple negative breast cancer cell line).

Further Western blot studies have shown that treatment with 25 µM (-)-oleocanthal increased the cleavage of caspase-8 and RIP, but not caspase-9 or cytochrome c. These results indicate that treatment with (-)-oleocanthal at 25 µM seems to be associated with caspase-8-dependent pathway, rather than mitochondrial stress, which results in activation of caspase-8, cleavage of RIP and caspase-3, leading to the proteolytic cleavage of PARP and activation of programmed cell death.

To further explore the role of c-Met in the survival of MDA-MB-231 cells; siRNA was used to specifically inhibit c-Met expression in these cells. Transfection of c-Met-targeted siRNA decreased c-Met protein expression by at least 90% (Figure 6C). The transfected adherent MDA-MB-231 cells exhibited a rounded-up phenotype starting after 24 h due to a mitotic arrest. Interestingly, c-Met depletion yielded a pattern of apoptosis that is remarkably similar to that following treatment with (-)-oleocanthal at 25 µM. Transfection of c-Met-targeted siRNA caused an increase in caspase-8 and RIP cleavage, with no effect on caspase-9 and cytochrome c levels (Figure 6D). These findings indicate that (-)-oleocanthal at 25 µM sensitizes cancer cells to induce caspase-8-dependent intrinsic apoptosis, at least in part, by downregulating c-Met expression in MDA-MB-231 human breast cancer cells. To further confirm the involvement of caspase-3 and caspase-8 activation in (-)-oleocanthal-induced apoptosis, MDA-MB-231 cells were pretreated with or without caspase family inhibitor Z-VAD-FMK. The results showed that 25 µM (-)-oleocanthal powerfully induces caspase-8 and caspase-3 activation and apoptosis in MDA-MB-231 cells, and caspase inhibitor Z-VAD-FMK treatment was able to completely inhibit the (-)-oleocanthal-induced apoptosis in MDA-MB-231 cells (Figure 6E).

### *In vivo* Antitumor Activity of (-)-oleocanthal

**Figure 7:**
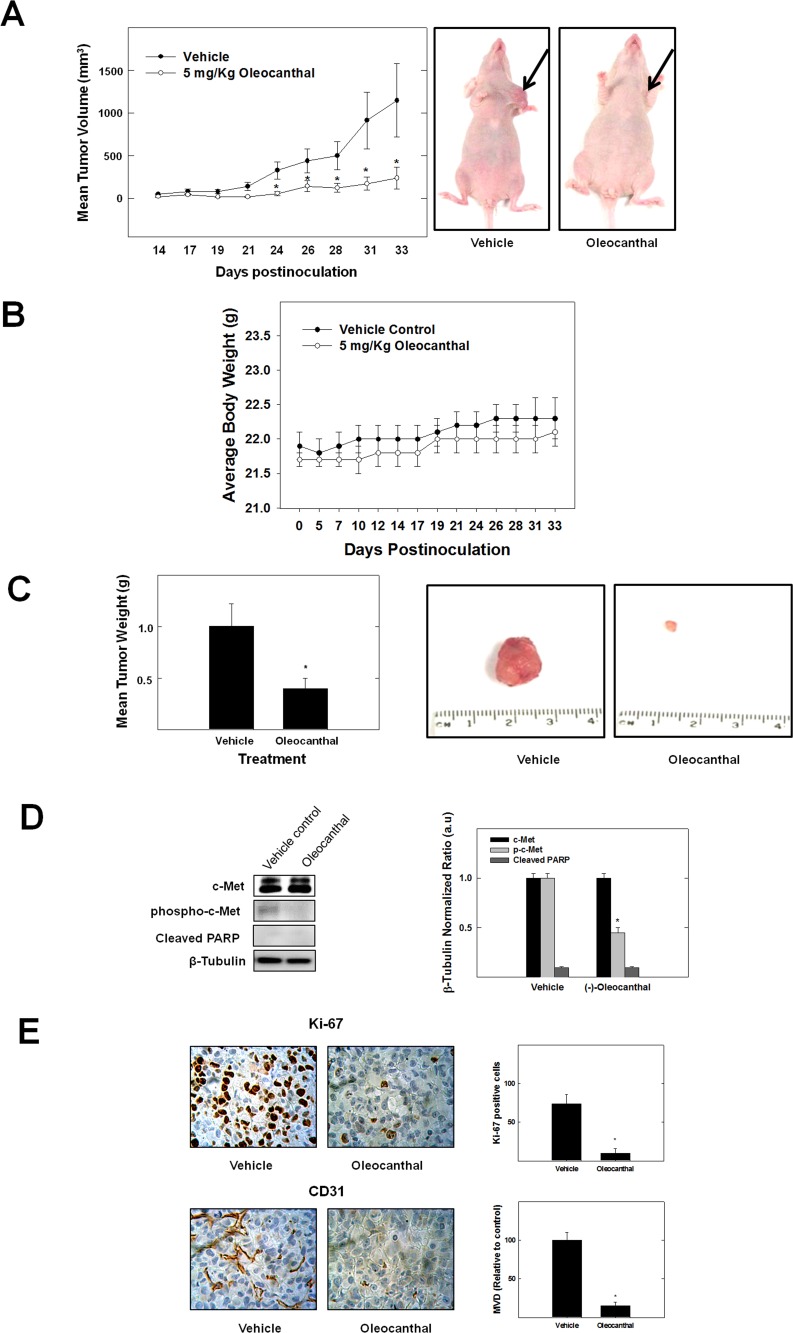
(-)-Oleocanthal treatment suppresses tumor growth in human tumor xenograft model. MDA-MB-231/GFP human breast cancer cells were cultured and resuspended in serum-free DMEM medium (20 µl). After anesthesia, cell suspensions (1×10^6^ cells/20 µl) were inoculated subcutaneously into the second mammary gland fat pad just beneath the nipple of each athymic nude mouse to generate orthotopic breast tumors. At 48 h post-inoculation, the mice were randomly divided into two groups: i) the vehicle-treated control group (n = 5), ii) the (-)-oleocanthal-treated group (n = 5). Treatment (3X/week) started 5 days postinoculation with intraperitoneal (i.p.) administered vehicle control or 5 mg/kg (-)-oleocanthal. (**A**) *Left panel*; tumor size was evaluated periodically during treatment at indicated days postinoculation. Tumor volume (V) was calculated by V = L/2 x W^2^, where L was the length and W was the width of tumors. Points, mean of tumor volume in mm^3^ of several tumors (n = 5) during the course of the treatment period; bars ± SEM. *P<0.05 as compared to vehicle-treated control. *Right panel*; shown are two mice harboring human breast cancer. The mouse on the right shows suppression of tumor growth with (-)-oleocanthal treatment (5 mg/kg/day) compared to vehicle treated control mouse on the left. (**B**) No significant change in body weight was observed among treated animals, indicating the safety of (-)-oleocanthal treatment. Error bars indicate SEM for n = 5. (**C**) Vertical bars indicate mean tumor weight at the end of the experiment (*left panel*). *P<0.05 compared to vehicle-treated controls. *Right panel* shows primary breast tumors from mice with vehicle-treated cancer (left), and cancer treated with (-)-oleocanthal (5 mg/kg/day) (right). (**D**) Protein expression of c-Met, phospho-c-Met and Cleaved PARP in breast tumors detected by Western blot. Scanning densitometric analysis was performed on all blots done in triplicate and the integrated optical density of each band was normalized with corresponding β-tubulin, as shown in bar graphs beside their respective Western blot images. Vertical bars in the graph indicate the normalized integrated optical density of bands visualized in each lane ± SEM, *P<0.05 as compared with vehicle-treated controls. (**E**) Immunostaining of sections (*left panel*) obtained from vehicle-treated or (-)-oleocanthal-treated (5 mg/kg/day) mice against Ki-67 (mitosis marker), CD31 (endothelial marker). *Right panel* shows quantification of Ki-67 positive cells and microvessel density (MVD). Ki-67+ cells in breast cancer tissues were examined in 5 areas at a magnification of ×200. Microvessel density (MVD) of breast tumor tissue sections was evaluated. Any CD31+stained endothelial cell or endothelial cell cluster was counted as one microvessel. The mean microvessel count of the five most vascular areas was taken as the MVD, which was expressed as the absolute number of microvessels per 1.485 mm^2^ (×200 field). Vertical indicate the average of 5 readings ± SEM, *P<0.05 as compared with vehicle-treated controls.

To test the antitumor activity of (-)-oleocanthal, orthotopic nude mouse model using MDA-MB-231/GFP human breast cancer cell line was used. In this experiment, 5 mg/kg (-)-oleocanthal caused a reduction in tumor growth by 60%, compared to vehicle-treated control group while it had no adverse effect on mice body weight or other clinical symptoms, indicating (-)-oleocanthal lacks potential systemic toxicity in athymic nude mice (Figures 7A–C). In addition, Western blot analysis of isolated tumor tissues showed relatively lower levels of phospho c-Met when compared to the vehicle treated control group without any change of total c-Met levels (Figure 7D). Moreover, there was no increase in cleaved PARP levels in the group of animals treated with (-)-oleocanthal suggesting that (-)-oleocanthal activity is mediated through cytostatic mechanisms rather than inducing apoptosis at the dose tested *in vivo* (Figure 7D). Furthermore, immunohistochemical analysis of tumor specimens showed that (-)-oleocanthal treatment suppressed mitosis and new vessel formation as evident by the suppression of the expression of their markers Ki-67 and CD31, respectively, compared to the vehicle-treated control group (Figure 7E). Tumor MVD calculated by new vessel formation using CD31 staining decreased significantly after (-)-oleocanthal treatment (Figure 7E).

## Discussion

Results of the present study demonstrated that (-)-oleocanthal treatment suppressed HGF-stimulated growth of human breast cancer cell lines MDA-MB-231, MCF-7 and BT-474 cells in dose- and time-dependent manners. Alternatively, (-)-oleocanthal treatment had little or no effect on the growth and/or viability of the non-tumorigenic human MCF10A mammary epithelial cells at a concentration which was many folds higher than the growth inhibitory concentration of the neoplastic breast cancer cells. In the highly metastatic MDA-MB-231 mammary cancer cells, (-)-oleocanthal treatment was also associated with induction of G1 cell cycle arrest. Further results showed that (-)-oleocanthal treatment caused a marked dose-dependent inhibitory effect on HGF-induced migration and invasion of MDA-MB-231 breast cancer cells *in vitro*. Remarkably, findings of animal studies showed that (-)-oleocanthal treatment resulted in a significant inhibition of tumor growth in an orthotopic model of breast cancer as compared to vehicle-treated control animals.

Breast cancer is not a single disease but is highly heterogeneous at both the molecular and clinical levels [24]. The molecular differences result in distinct clinical outcomes and responses to treatment [25]. Recently, strong evidence supports the role for the hepatocyte growth factor (HGF) and its receptor in the development and progression of breast carcinoma [26]. Under physiological conditions, HGF regulates epithelial development and morphogenesis in different organs [27]. In the human breast, HGF is produced primarily in the mammary stroma, whereas c-Met is expressed in the epithelium [28]. HGF-mediated activation of c-Met results in a complex genetic program referred to as “invasive growth”, consisting of a series of physiological processes, including cell proliferation, motility, invasion, angiogenesis, and branching tubulogenesis [29]. Results of this study showed that HGF stimulated the growth of multiple mammary epithelial carcinoma cell lines *in vitro*. HGF induced activation and phosphorylation of c-Met in MDA-MB-231, MCF-7 and BT474 breast cancer cells in culture. Treatment with (-)-oleocanthal resulted in a dose- and time-dependent inhibition of the growth of mammary cancer cells *in vitro*. The inhibition of mammary cancer cell growth was associated with the ability of (-)-oleocanthal treatment to block c-Met receptor activation in response to its natural ligand HGF in MDA-MB-231, MCF-7, and BT474 cancer cell lines in culture. In addition, the antiproliferative activity of (-)-oleocanthal was accomplished in cancer cells maintained in the presence of HGF as well as in HGF-free treatment media. Interestingly, concentrations of (-)-oleocanthal required to induce 50% inhibition of cancer cell growth were greater for breast cancer cells maintained in HGF-free media as compared to those maintained in media supplied with HGF. While this finding indicated that (-)-oleocanthal treatment is more effective in the presence of HGF, confirming a direct inhibition of the HGF/c-Met axis, these results also suggest other potential mechanisms for the antiproliferative effects of (-)-oleocanthal that may not be mediated through a direct suppression of the HGF/c-Met signaling pathway. Furthermore, (-)-oleocanthal treatment was shown to have no remarkable effect on the viability and growth of non-tumorigenic MCF10A mammary epithelial cells in culture. Thus, (-)-oleocanthal treatment can achieve significant antiproliferative effects with concentrations that have no or little effect on the viability of the non-tumorigenic mammary epithelial cells.

The biological functions of the HGF/c-Met signaling are mediated through a variety of downstream effectors. The results of this study showed that activation of the HGF/c-Met signaling pathway in MDA-MB-231 mammary cancer cells resulted in the activation of downstream effectors Akt and MAPK. Exposure to growth inhibitory concentrations of (-)-oleocanthal blocked HGF-induced phosphorylation and activation of Akt and MAPK in MDA-MD-231 mammary cancer cells. It is well-established that HGF/c-Met signaling for mitogenesis and growth occurs through the MAPK signaling pathway [30], [31]. In addition, activation of c-Met prevents apoptosis and maintains cancer cell survival through activation of PI_3_K and subsequent Akt-NFκB activation [32], [33]. Accordingly, (-)-oleocanthal treatment effectively blocked growth and mitogenesis through suppression of HGF-induced c-Met activation and subsequent activation of downstream effectors. During mitogenesis, progression through the cell cycle is a highly organized and regulated process. Cells must pass through a restriction point in the G1 phase before progression into S phase and subsequently undergoing mitosis [34]. Early in G1 phase progression, mitogenic factors enhance the expression of cyclin D1. CDK4/6 are activated by binding to cyclin D1 and these cyclin/CDK complexes consequently phosphorylate (inactivate) the cell cycle restriction protein, retinoblastoma (Rb), thereby releasing E2F transcription factors and leading to the transcription of genes required for progression through the S phase [34]. Upstream inhibitors such as p21 and p27 alter the activity of the CDK-cyclin complexes [34]. (-)-Oleocanthal treatment was shown to reduce expression of cyclin D1 and CDK6, and caused a corresponding increase in p21 and p27 levels in MDA-MB-231 breast cancer cells. Thus, (-)-oleocanthal treatment is associated with cytostatic activity and G1 cell cycle arrest, the findings that contribute to the growth inhibitory activity of this compound in mammary tumors. It is well established that blockade of cell cycle progression can initiate programmed cell death in a number of tumor cell types. Results of the present study showed that higher concentration of (-)-oleocanthal treatment induced apoptosis in MDA-MB-231 cells. Cytotoxic activity of (-)-oleocanthal was initiated by activation of caspase-8 and cleavage of RIP and caspase-3, leading to the proteolytic cleavage of PARP. It has been reported that death domain kinase (RIP) is cleaved by cleaved caspase-8 in death receptor-mediated apoptosis [35]. Apoptosis of MDA-MB-231 cells by (-)-oleocanthal was associated with a marked reduction of the total c-Met protein expression. These findings indicate that c-Met protects breast cancer cells from apoptosis and it contributes to their survival. (-)-Oleocanthal treatment caused apoptosis of breast cancer cells by downregulating c-Met.

HGF is able to induce epithelial cell dissociation and scattering. Earlier studies have shown HGF to act as a motogen or morphogen in most breast carcinoma cell lines [36]. In order for epithelial cells to ‘scatter,’ the attenuation of cell-cell adhesions is a prerequisite [37], [4]. HGF-induced scattering and motility is a tightly controlled process mediated by multiple effectors including Rac, Rho, and Brk [6], [31], [37]. Results of this study showed that (-)-oleocanthal treatment effectively suppressed HGF-induced migration of MDA-MD-231 in wound healing assay. Similarly, (-)-oleocanthal blocked HGF-induced invasion of the highly invasive MDA-MB-231 breast cancer cells. The antimigratory and anti-invasive activities of (-)-oleocanthal were associated with suppression of activation of Brk, paxillin, and Rac1 in response to HGF stimulation in MDA-MB-231 cancer cells. These findings are of particular significance taking into consideration that c-Met-mediated invasive growth plays an important role in the development of the more aggressive and metastatic phenotypes of breast cancer. Epithelial-to-Mesenchymal Transition (EMT) is considered to be the first step in the metastatic cascade of carcinoma cells [38]. In EMT, epithelial cells lose cell-cell contacts and apical-basal polarity, and acquire mesenchymal phenotype [39]. It has been proposed that EMT is involved in cancer progression, particularly during invasion, intravasation and migration. E-cadherins are a family of transmembrane glycoproteins that mediate cell-cell adhesion. E-cadherin is expressed in most epithelial cells, and it is primarily responsible for the initial adhesion of these cells and also promotes polarity. Analysis of many epithelial cancers suggested that loss of E-cadherin correlates with tumor cell invasion [39]. Vimentin is an intermediate filament protein normally expressed in cells of mesenchymal origin [40]. Vimentin regulates cell migration in many cell types [40]. It has been reported that the loss of E-cadherin causes disruption of cell adhesion and polarity allowing tumor cell metastasis, while the translocation of β-catenin into the nucleus might be required to induce the expression of genes that promote cell proliferation and invasion [39]. Eventually, epithelial cells that undergo EMT lose their epithelial cell characteristics to acquire a mesenchymal phenotype and become migratory and invasive [41]. c-Met is a key promoter of EMT [38]. Previous studies showed that sustained activation of HGF/c-Met signaling is associated with dissociation of cadherin-based adherens junctions, followed by loss of E-cadherin expression [42], [43]. Based on our study, (-)-oleocanthal treatment restored the expression of the epithelial markers E-cadherin and Zo-1 in MDA-MB-231 and suppressed the expression of the mesenchymal marker vimentin. Alternatively, (-)-oleocanthal treatment stabilized the expression E-cadherin and Zo-1 in MCF-7 and BT-474 breast cancer cells. Therefore, these findings suggested that (-)-oleocanthal treatment caused a marked reduction in mammary cancer cell scattering, motility, and invasion proposing a potential role of (-)-oleocanthal in stabilizing cell-cell adhesion. *In vivo* characterization of (-)-oleocanthal treatment showed a potent inhibition of MDA-MB-231 xenograft growth in female athymic nude mice. (-)-Oleocanthal administration in experimental animals resulted is suppression of tumor growth as compared to control animals. These findings were associated with decreased cancer cell proliferation as indicated by a reduction of Ki-67 and CD31 staining in treated animals. In agreement with cellular data, MDA-MB-231 tumors from (-)-oleocanthal-treated mice revealed a marked decrease in c-Met phosphorylation in comparison with those of control mice.

Given the oncogenic role of aberrant HGF/c-Met signaling, c-Met has become an attractive therapeutic target. Several different strategies are being explored to reach this goal, including the development of competitors of HGF/c-Met, monoclonal antibodies directed against HGF and c-Met, inhibitors of c-Met expression, and small-molecule tyrosine kinase inhibitors directed against c-Met [4], [33]. Whereas some of these approaches are better suited to block ligand-mediated c-Met activity, small molecule kinase inhibitors offer the most versatile approach by inhibiting HGF-dependent tumors as well as tumors driven by other c-Met-dependent mechanisms, such as receptor amplification and activating mutations [37], [44]. Earlier studies identified (-)-oleocanthal as potential inhibitor for the c-Met kinase domain [17]. However, a growing evidence in literature shows that the potential anticancer mechanisms of (-)-oleocanthal are not limited to its c-Met inhibitory activity. Clearly, (-)-oleocanthal exerts a remarkable anti-inflammatory activity [10]. The anti-inflammatory activity of oleocanthal is mediated through inhibition of macrophage inflammatory protein 1- alpha (MIP-1 α) and interleukin-6 (IL-6) expression and secretion [13], [45]. Recently, oleocanthal anti-inflammatory activity has been also associated with inhibition of 5-lipoxygenase, an enzyme which catalyzes the initial steps in the biosynthesis of pro-inflammatory leukotrienes [46]. Additionally, oleocanthal demonstrated a potent inhibitory activity of heat shock protein 90 (Hsp90), an essential molecular chaperone involved in different cancer hallmarks [47]. Investigations by Margarucci and colleagues revealed ATPase activity inhibition and changes in the Hsp90 oligomerization state promoting the loss of Hsp90 molecular chaperone function upon treatment with oleocanthal [47]. Moreover, the knowledge available on the metabolic fate, absorption and bioavailability of (-)-oleocanthal is still unclear [48]. Accordingly, the molecular mechanism of action of (-)−oleocanthal is being a subject of investigation recently. Clearly, (-)-oleocanthal interferes with multiple pathways in cancer. These findings might explain, at least in part, the significant *in vivo* activity of (-)-oleocanthal in this study.

(-)-Oleocanthal, an olive-oil phenolic component, has captured an increasing interest in the elucidation and characterization of its potential anticancer activity. Collectively, the present findings of this study promoted (-)-oleocanthal from hit to lead status for the control of breast cancer. (-)-Oleocanthal reduced c-Met kinase activity, cell growth, migration, and invasion of breast cancer cells. In addition, (-)-oleocanthal induced G1 cell cycle arrest and apoptosis, as well as inhibited c-Met-dependent signaling in cultured breast cancer cells and tumorigenicity in mouse model. These findings further promote (-)-oleocanthal as a promising lead with potential therapeutic use as a dietary supplement for the control of c-Met-dependent malignancies.
